# Effect of Extraction Methods on the Physicochemical Properties, Chemical Composition, and Antioxidant Activities of Samara Oil

**DOI:** 10.3390/foods12173163

**Published:** 2023-08-23

**Authors:** Xiujuan Li, Mimi Guo, Yalin Xue, Zhangqun Duan

**Affiliations:** Institute of Cereal & Oil Science and Technology, Academy of National Food and Strategic Reserves Administration, Beijing 100037, China; lixj@ags.ac.cn (X.L.); gmm@ags.ac.cn (M.G.); xyl@ags.ac.cn (Y.X.)

**Keywords:** samara oil, solvent extraction, mechanical extraction, physicochemical properties, oxidative stability index

## Abstract

Samara oil (*Elaeagnus mollis* Diels kernel oil) exhibits diverse healthy functions; however, the effect of extraction on its quality is still unclear. The present study was undertaken to evaluate the effect of extraction methods (solvent extraction: ethyl acetate, acetone, *n*-hexane, and petroleum ether; mechanical extraction: hot-pressing and cold-pressing) on the color, acid value, peroxide value, fatty acid composition, bioactive compounds, antioxidant activities, and oxidative stability index of samara oil obtained from *Elaeagnus mollis* Diels kernels. The results indicated that extraction methods affected the physicochemical properties, chemical composition, and antioxidant activities of samara oil except for fatty acid composition and γ-tocopherol. The highest values of bioactive compounds including polyphenols (140.27 mg gallic acid equivalent (GAE)/kg) and carotenoids (42.95 mg/kg) were found in samara oil extracted with acetone. The values of 2,2-diphenyl-1-picrylhydrazyl (DPPH) and 2,2′-azino-bis(3-ethylbenzothiazoline-6-sulfonic acid) diammonium salt (ABTS) assays, as well as oxidative stability index (OSI), were the highest in this oil. Correlation analysis results demonstrated that DPPH, ABTS, and OSI of samara oil were positively correlated with polyphenols and carotenoids. After evaluation, acetone could be used to extract samara oil. The study provides new information on the samara oil process.

## 1. Introduction

**Figure 1:**
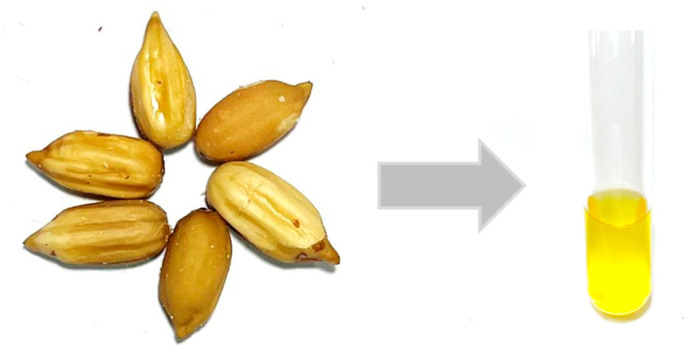
*Elaeagnus mollis* Diels (*Elaeagnus* L.) and its oil.

*Elaeagnus mollis* Diels (*Elaeagnus* L.) belongs to the family of Elaeagnaceae and is one of the most important economical and ecological plants in the mountainous regions (altitude, 800–1500 m) of Shanxi Province in China [1]. According to Mu et al. [1], its seed contains 46%–51% of crude oil and 26%–33% of crude protein. The oil made from its kernel is samara oil (Figure 1).

The high oil yield is very important to the oil industry. On the other hand, the higher recovery of bioactive compounds is also the main aim for oil processing. For these reasons, many researchers focus on the increase in the oil yield and the higher recovery of these compounds and develop corresponding methods to extract them from oilseeds. Soxhlet extraction method is commonly and widely used for oil extraction because of its good economic benefits and easy operation. However, this method exhibits many disadvantages including long duration time and heating [2]. Hot-pressing technology is a time-honored oil extraction method. Before pressing, the oilseed is roasted for a certain period of time, which gives the oilseed and oil pleasant flavors such as roasted, nutty, and sweet flavors for increasing consumer desire [3,4]. Cold-pressing technology is prevalent in recent years due to its natural fresh flavor and high amounts of bioactive compounds [3]. Additionally, some emerging technologies, including ultrasonic-assisted extraction, microwave-assisted extraction, supercritical carbon dioxide (CO_2_) extraction, and subcritical extraction methods, have been investigated by scientists for high efficiency oil extraction [2,5,6,7].

Recently, a few articles reported the effect of extraction methods on the quality of samara oil. For instance, Mu et al. [1] investigated and compared Soxhlet and supercritical carbon dioxide extraction methods on the oil yield, fatty acid composition, and triglyceride profile of samara oil. The authors found that supercritical carbon dioxide could be a better option for samara oil extraction due to its higher recovery of bioactive constituents [1]. Wu et al. [8] compared fatty acid composition, phytosterol, and tocopherol components of four varieties of oilseeds including *Elaeagnus mollis* Diels (samara oil), *Xanthoceras sorbifolium* Bunge, *Amygdaluspedunculata* Pall., and *Paeonia suffruticosa* Andr. based on the Soxhlet extraction method. However, the effect of different solvent and mechanical pressing methods for the extraction of samara oil has not been reported. The main aim of this study was to measure the effect of extraction methods including solvent and mechanical methods on the color, acid value, peroxide value, fatty acid composition, bioactive constituents (polyphenols, carotenoids, and tocopherols), antioxidant activities (DPPH and ABTS), and oxidative stability index (OSI) of samara oil obtained from the kernel of *Elaeagnus mollis* Diels by titration, spectroscopy, and chromatography methods. The study provides new information on the samara oil process.

## 2. Materials and Methods

### 2.1. Reagents

Chromatographic grade heptane, tetrahydrofuran, methanol, isooctane, isopropanol, ethyl acetate, acetone, *n*-hexane, and acetic acid were purchased from Fisher (Shanghai, China). Additionally, (±)-6-hydroxy-2,5,7,8-tetramethylchromane-2-carboxylic acid (97%), 2,2-diphenyl-1-picrylhydrazyl (95%), 2,2′-azino-bis(3-ethylbenzothiazoline-6-sulfonic acid) diammonium salt (98%), γ-tocopherol, and gallic acid were obtained from Sigma-Aldrich (Shanghai, China). All other reagents including petroleum ether (boiling point: 30 °C–60 °C) were of analytical grade.

### 2.2. Elaeagnus mollis Diels Kernels Preparation

Ten kilograms of *Elaeagnus mollis* Diels fruits were collected from five individual trees from a local farm in Xiangning County, Shanxi Province of China in the harvesting season of 2020. Then, the fruits were immediately dried at 40 °C until the moisture was less than 6%, and the pericarps were removed manually. The dried kernels of *Elaeagnus mollis* Diels were sealed in the plastic Ziplock bags and stored at 4 °C before samara oil extraction.

### 2.3. Solvent Extraction

The solvent extraction of samara oil followed the method described by other researchers with minor modification [2]. Before solvent extraction, the kernels of *Elaeagnus mollis* Diels were ground using a Foss Knifetec 1095 grinder (Foss, Hilleroed, Denmark). The kernel powder was extracted using ethyl acetate, acetone, *n*-hexane, and petroleum ether at the ratio of 1:3 (*w*/*v*) with a 06HT ultrasonic cleaner (Dongguan Jiajin machinery Co., Ltd., Dongguan, China) for 30 min immediately. Then, the mixture was centrifuged by an Eppendorf 5430R centrifuge (Eppendorf, Hamburg, Germany) at 5000 rpm for 5 min, and the upper layer was collected for solvent evaporation. This procedure was conducted twice. The supernatant was combined and evaporated at 45–50 °C. The particles in samara oil were discarded after passing through the filter paper (opening size: 30–50 μm). Finally, clear oils were collected and stored in brown glass bottles at 4 °C for further analysis. Each solvent extraction was repeated in triplicate.

### 2.4. Mechanical Extraction

The mechanical extraction of samara oil was performed using the method published elsewhere with slight modification [3,4]. For hot-pressing, the kernels of *Elaeagnus mollis* Diels were heated at 120 °C for 30 min using QYHX-1505A automatic oven (Shanghai Qiyi Industrial Equipment Co., Ltd., Shanghai, China). After heating, the kernels were immediately fed in a self-made oil expeller to obtain samara oil. For cold-pressing, the kernels of *Elaeagnus mollis* Diels were directly pressed without pre-heating. The impurities of hot-pressing and cold-pressing oils were removed by passing through the filter paper. Finally, clear oils were collected and stored in brown glass bottles at 4 °C for further analysis. Each mechanical extraction was repeated in triplicate.

### 2.5. Color Determination

The color (L* (lightness), a* (greenness to redness), and b* (blueness to yellowness) values) of samara oil obtained by solvent and mechanical extraction were performed according to the method reported by Zhang et al. [9].

### 2.6. Acid Value (AV) and Peroxide Value (PV)

The physicochemical characteristics including acid value (AV) and peroxide value (PV) of samara oil were examined following the methods published elsewhere [10].

### 2.7. Fatty Acid Composition

The preparation of fatty acid methyl esters were performed by the methylation of samara oil following the AOCS method [10]. The separation of fatty acid methyl esters was conducted on an Agilent 6890A gas chromatography combined with a hydrogen flame ionization detector (Agilent, Santa Clara, CA, USA) and a VF-23MS capillary column (30 m × 0.25 mm i.d., 0.25 μm film thickness; Agilent, Santa Clara, CA, USA). High-purity nitrogen (99.999%) was used as carrier gas at a constant flow of 1 mL/min. The split ratio was 100:1, and the injection volume was 1 μL. The injection and detector temperatures were set at 260 °C. The temperature program was as follows. The initial temperature was 110 °C for 3 min, then increased to 220 °C at a rate of 4 °C/min, and held for 15 min at 220 °C. The peaks of fatty acid methyl esters were identified using their corresponding standards such as palmitic, stearic, oleic, linoleic, and linolenic acid. The results were expressed as a percentage of the oil.

### 2.8. Polyphenols

The extraction and determination of polyphenols of samara oil investigated in this study were performed using the method described by Zhang et al. [11]. Briefly, 2 g of samara oil were weighed and extracted with 5 mL of methanol for 2 min. The supernatant was collected and stored at 4 °C prior to analysis. The Folin–Ciocalteu colorimetric method was used for the measurement of polyphenols in the samara oil. The methanol extract of samara oil (1 mL), 7.5% sodium carbonate solution (2 mL), and Folin–Ciocalteu reagent (0.5 mL) were mixed and shaken for 2.5 min. The volume of the solution was 10 mL. Then, the solution was incubated at 70 °C for 30 min and cooled down to room temperature. The absorbance at 765 nm was measured.

### 2.9. Carotenoids

The content of carotenoids was determined using a Perkin Elmer Lambda 45 UV/VIS spectrophotometer (Perkin Elmer, Waltham, MA, USA). Briefly, 0.5 g of samara oil was dissolved in 5 mL of *n*-hexane and mixed thoroughly. The absorbance of solution was determined at 446 nm. The calculation of carotenoids followed the recently published article [11].

### 2.10. Tocopherols

Tocopherols in samara oil were determined according to the method reported elsewhere [12]. An amount of 0.1 g of Samara oil was dissolved in 10 mL of heptane. The solution was mixed thoroughly and passed through a 0.22 μm nylon membrane. The analysis of tocopherols in samara oil was conducted on a Waters e2695 high performance liquid chromatography (Waters, Singapore). The excitation and emission wavelength of a fluorescence detector were set as 295 and 330 nm, respectively. Tetrahydrofuran and heptane were used as the mobile phase at the ratio of 40:1000 (*v*/*v*). The flow rate of the mobile phase was 1 mL/min.

### 2.11. Antioxidant Activities

DPPH and ABTS assays of samara oil extracted using solvent and mechanical methods were measured following a previous study [11]. The preparation of oil extract was the same as polyphenols. For DPPH assay, 100 μL of the methanol extracts, 1400 μL of methanol, and 500 μL of DPPH methanol solutions were mixed and left in the dark for 15 min. The absorbance was measured at 517 nm. For ABTS assay, before measurement, the ABTS^•+^ solution was placed in the dark for 16 h and then diluted with methanol to the absorbance of 0.70 at 734 nm. An amount of 0.02 mL of the methanol extracts, 0.08 mL of methanol, and 2.40 mL of ABTS^•+^ solution was mixed and incubated at 30 °C for 5 min. The absorbance was measured at 734 nm.

### 2.12. Oxidative Stability Index

The oxidative stability index (OSI) of samara oil extracted with solvent and mechanical methods was measured using an Omnion OSI-24 Rancimat apparatus (Omnion, Rockland, MA, USA) based on the official method [10]. Three g of samara oil were weighed in the reaction tube and then heated at 110 °C with an air flow of 10 L/h. Inducing time was automatically calculated.

### 2.13. Statistical Analysis

All the quantitative analyses were performed in duplicate. Data collected from the study were illustrated as mean ± standard deviation. One-way ANOVA (Duncan’s test) and Pearson correlation analysis were performed to evaluate the effect of extraction methods on the chemical composition and antioxidant activities, as well as their correlation to samara oil using SPSS 16 (SPSS, Chicago, IL, USA) and Origin Pro 2022b software (OriginLab, Northampton, MA, USA).

## 3. Results and Discussion

### 3.1. Color

The color values (L*, a*, and b*) of samara oil obtained from *Elaeagnus mollis* Diels kernels through solvent and mechanical extraction methods are illustrated in Table 1. The values in lightness (L*) of samara oil using solvent extraction ranged from 27.24 (*n*-hexane) to 31.03 (acetone), indicating that samara oil extracted with acetone exhibited the highest lightness. The lightness values of the other three samara oil extracts with organic solvents were similar (27.24–28.17). Hot-pressing and cold-pressing samara oil showed near lightness (26.72–27.50), lower than the solvent extraction method. The a* values of samara oil were −0.48–0.00 for solvent extraction and 0.23–0.28 for mechanical extraction. Samara oil extracted with petroleum ether had the highest b* value (10.14), followed by cold-pressing, hot-pressing, *n*-hexane, and acetone; ethyl acetate showed the lowest b* value (6.24). Samara oil extracted by cold-pressing and hot-pressing methods suggested similar b* values. All these results demonstrated that extraction methods affected the color of samara oil. Chouaibi et al. found similar results that extraction methods could affect the color of red pepper seed oils [13]. Not only that, in our previous study, we found the color darkened significantly as the roasting temperature and time increased [9].

### 3.2. Acid Value and Peroxide Value

Acid value (AV) and peroxide value (PV) are the two most important parameters for reflecting the quality of oils. The AV determines the free fatty acids in the oils due to the hydrolysis of glycerides, and the PV measures the formation of primary oxidation products of oils [14,15]. The AV of samara oil obtained through solvent extraction and mechanical extraction methods were 0.66–1.33 mg KOH/g oil (Table 1). Among them, samara oil extracted with acetone had the highest AV, followed by ethyl acetate, petroleum ether, n-hexane, hot-pressing, and cold-pressing. The AV of samara oil obtained using the mechanical extraction method was lower than that obtained using the solvent extraction method. This could be explained by the higher extraction ability of solvents than mechanical force [16]. The PV of samara oil obtained using different extraction methods was less than 1 mmol/kg except for ethyl acetate extraction (1.11 mmol/kg). All the AV and PV of samara oil investigated in this study were less than the recommended values of 4 mg KOH/g for AV and 5 mmol/kg for PV in the crude oils by FAO/WHO [17].

### 3.3. Fatty Acid Composition

The fatty acid composition of samara oil extracted from *Elaeagnus mollis* Diels kernels by solvent and mechanical extraction methods was investigated in this study (Table 2). Seven fatty acids containing palmitic (C16:0), stearic (C18:0), oleic (C18:1), linoleic (C18:2n6), linolenic (C18:3n3), arachidic (C20:0), and eicosenoic acid (C20:1) were determined. Among them, linoleic acid (C18:2n6) was the highest (48.07%–48.28%), followed by oleic (C18:1, 36.78%–37.14%), linolenic (C18:3n3, 7.37%–7.57%), palmitic (C16:0, 4.09%–4.14%), and stearic acid (C18:0, 2.60%–2.65%). The amounts of polyunsaturated, monounsaturated, and saturated fatty acids were 55.54%–55.74%, 37.32%–37.57%, and 6.89%–6.94%, respectively. This observation was in line with the result reported by Mu et al. [1]. The amounts of oleic and linoleic acid of samara oil were similar to that of sunflower oil [18]. Importantly, samara oil contained 7.37%–7.57% of linolenic acid, similar to another one of the healthy vegetable oils, walnut oil [19]. The amount of saturated fatty acid (SFA) of samara oil was less than 7%, and the sum of monounsaturated fatty acid (MUFA) and polyunsaturated fatty acid (PUFA) was higher than 92%. Singh and Kumar investigated the effect of various extraction methods consisting of cold-pressing extraction, Soxhlet extraction, and mechanical shaking extraction on fatty acid composition of pumpkin seed oil, and the results showed no obvious variation between the extraction methods [20]. Similarly, Liu et al. found that extraction methods did not influence fatty acid composition of pomegranate seed oil [21].

### 3.4. Polyphenols

Polyphenols are found in many plants including oilseeds and have been reported as antioxidants in many studies [21]. As seen in Table 3, polyphenols in samara oil obtained by solvent and mechanical extraction methods were measured in this study. Samara oil extracted with acetone had the highest amount of polyphenols, up to 140.27 mg GAE/kg, far higher than the other extraction methods. The content of polyphenols in samara oil extracted with ethyl acetate was 38.01 mg GAE/kg, only less than acetone. In this study, the order of the polarity of the solvent used was as follows: acetone > ethyl acetate > *n*-hexane > petroleum ether. Polyphenols were polar compounds due to their phenolic hydroxyl group [22]. Therefore, the highest amount of polyphenols in samara oil extracted with acetone could be explained by the principle of “like dissolves like”. Samara oil extracted with hot-pressing and cold-pressing methods had similar polyphenol contents (23.36–24.52 mg GAE/kg).

### 3.5. Carotenoids

Up to now, over 700 carotenoids have been found in nature. According to the structure, carotenoids have been divided into two categories, carotenes and xanthophylls [23]. Carotenes are a group of compounds with C40 tetraterpene structures, consisting of eight isoprenoids, and have been widely studied and used as natural antioxidants [23]. In this study, the content of carotenoids, especially carotene, in samara oil extracted with acetone was the highest (42.95 mg/kg). The high amount of carotenoids in samara oil was extracted with acetone due to its powerful dissolution ability (Table 3). The amount of polyphenols in samara oil extracted with solvent and mechanical extraction methods was near except for acetone extraction.

### 3.6. Tocopherols

Tocopherols, also called vitamin E, are the major antioxidants in plants including oilseeds, and their amount varies largely in different oilseeds. Meanwhile, tocopherols play important roles in the balance between oxidation and antioxidation in organisms [24]. Tocopherols contain four types including α-, β-, γ-, and δ-tocopherol [25]. As illustrated in Table 3, only γ-tocopherol was identified in samara oil extracted with solvent and mechanical methods, and its amount varied from 669.98 to 722.05 mg/kg, lower than the results reported by Wu et al. [8], who found that the content of γ-tocopherol in samara oil made from *Elaeagnus mollis* Diels kernels was 109.58 mg/100 g. This may be due to a different variety or environment. The amount of γ-tocopherol from different extraction methods showed no obvious difference.

### 3.7. Antioxidant Activities

The DPPH and ABTS free radical scavenging assays were applied to analyze the antioxidant activities of samara oil extracted with solvent and mechanical methods. DPPH radicals have a single electron and a strong absorption at 517 nm. The absorption gradually reduces when a free radical scavenger is present due to its single electron coordination pair. The reaction of ABTS with K_2_S_2_O_8_ generates stable free radical ABTS^•+^, which has the maximum absorption at 734 nm. When the free radical is removed, the solution color becomes lighter [26]. As shown in Table 4, samara oil extracted with acetone showed the highest DPPH and ABTS values, 817.30 and 1238.60 μmol Trolox equivalent (TE)/100 g, respectively, followed by ethyl acetate. The DPPH and ABTS values of samara oil using mechanical extraction methods were lower than using solvent extraction methods. A similar result was observed by Chouaibi et al., who found the DPPH and ABTS values of red pepper seed oils extracted using the Soxhlet method were higher than that by using cold pressing [13].

### 3.8. Oxidative Stability Index

The oxidative stability index (OSI) was employed for determining the oxidative stability of samara oil extracted using solvent and mechanical methods. The OSI mainly examines the primary or secondary oxidation products in vegetable oils [27]. The high value of OSI means high antioxidant activities, depending on the content and efficacy of antioxidants and the degree of unsaturation of fatty acids [27]. For example, the OSI of peanut oil (6.02–13.93 h) was higher than flaxseed oil (0.52–2.38 h) due to flaxseed oil containing a higher amount of polyunsaturated fatty acid, linolenic acid (54%) [15,27]. Additionally, the addition of antioxidants in the oils increased their oxidative stability index [28]. In this study, the values of OSI ranged from 6.26 to 21.38 h (Table 4). Samara oil extracted with acetone exhibited the highest OSI value at 21.38 h, which agreed with the results of the polyphenols and carotenoids investigated in this study. The OSI values of samara oil extracted with ethyl acetate, *n*-hexane, petroleum ether, and mechanical extraction methods showed an insignificant difference.

### 3.9. Correlation Analysis

**Figure 2:**
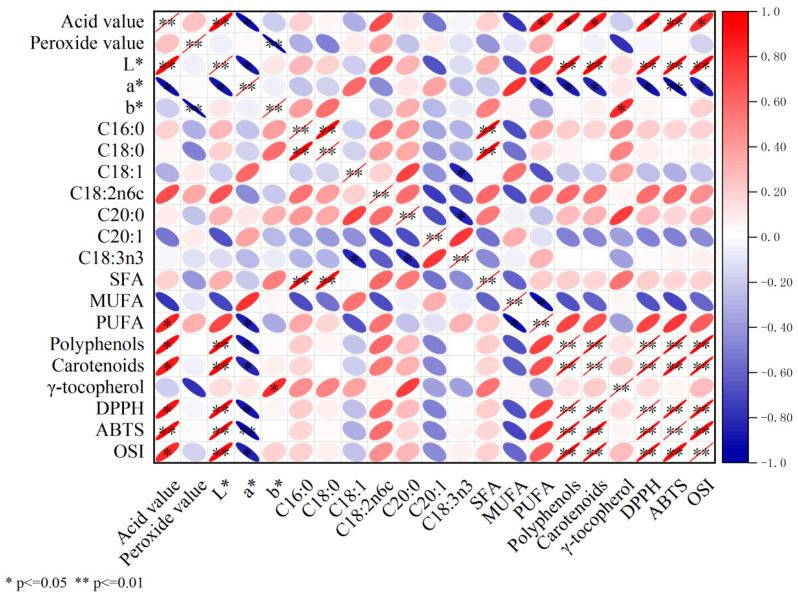
The correlation between the different parameters of samara oil obtained from *Elaeagnus mollis* Diels kernels extracted by solvent and mechanical methods. Red means positive correlation and blue means negative correlation; the scale ranged from −1 to 1; *, *p* ≤ 0.05 (significant); **, *p* ≤ 0.01 (highly significant); L*, lightness; a*, greenness to redness; b*, blueness to yellowness; C16:0, palmitic acid; C18:0, stearic acid; C18:1, oleic acid; C18:2n6, linoleic acid; C18:3n3, linolenic acid; C20:0, arachidic acid; C20:1, eicosenoic acid; SFA, saturated fatty acid; MUFA, monounsaturated fatty acid; PUFA, polyunsaturated fatty acid; DPPH, 2,2-diphenyl-1-picrylhydrazy; ABTS, 2,2′-azino-bis(3-ethylbenzothiazoline-6-sulfonic acid) diammonium salt; OSI, oxidative stability index.

The correlation coefficients between physicochemical properties, chemical composition, and antioxidant activities and the oxidative stability index of samara oil extracted with solvent and mechanical methods were investigated and calculated, and the results are illustrated in Figure 2. The DPPH, ABTS, and OSI of samara oil were positively correlated with acid value, L* value, polyphenols, and carotenoids, while negatively correlated with a* value. Insignificant correlation was observed between antioxidant activities (DPPH, ABTS, and OSI) and γ-tocopherol of samara oil. The finding demonstrated that oxidative stability of samara oil was not related to γ-tocopherol. The a* value was negatively correlated with PUFA, polyphenols, carotenoids, DPPH, and ABTS. The high amounts of polyphenols and carotenoids were mainly responsible for the high oxidative stability of samara oil.

## 4. Conclusions

In the present study, the effect of solvent and mechanical extraction methods on the physicochemical properties, chemical composition, and antioxidant activities of samara oil obtained from *Elaeagnus mollis* Diels kernels was investigated for the first time. The extraction method affected the color, AV, PV, polyphenols, carotenoids, DPPH, ABTS, and OSI of samara oil. Fatty acid composition and γ-tocopherol showed no obvious difference. Importantly, samara oil extracted with acetone exhibited the highest amount of bioactive compounds including polyphenols and carotenoids, subsequently leading to the highest DPPH, ABTS, and OSI. Therefore, acetone could be considered as the optimal solvent for samara oil extraction. The study provides new insight into the understanding of the extraction of samara oil and the possibility that acetone will be used as the extraction solvent for the processing of samara oil in the future.

## Figures and Tables

**Table 1:** Color, acid value, and peroxide value of samara oil extracted by solvent and mechanical methods.

Parameters	Solvent Extraction				Mechanical Extraction	
	Ethyl Acetate	Acetone	*n*-Hexane	Petroleum Ether	Hot-Pressing	Cold-Pressing
L*	27.82 ± 0.01 ^d^	31.03 ± 0.01 ^f^	27.24 ± 0.01 ^b^	28.17 ± 0.02 ^e^	27.50 ± 0.00 ^c^	26.72 ± 0.01 ^a^
a*	0.00 ± 0.00 ^c^	−0.48 ± 0.01 ^a^	−0.08 ± 0.05 ^b^	0.00 ± 0.00 ^c^	0.23 ± 0.01 ^d^	0.28 ± 0.06 ^d^
b*	6.42 ± 0.07 ^a^	9.18 ± 0.04 ^c^	9.17 ± 0.01 ^c^	10.41 ± 0.01 ^d^	9.04 ± 0.02 ^b^	9.01 ± 0.00 ^b^
Acid value (mg KOH/g)	1.02 ± 0.02 ^e^	1.33 ± 0.07 ^f^	0.81 ± 0.01 ^c^	0.86 ± 0.01 ^d^	0.77 ± 0.01 ^b^	0.66 ± 0.02 ^a^
Peroxide value (mmol/kg)	1.11 ± 0.03 ^e^	0.55 ± 0.01 ^c^	0.47 ± 0.00 ^b^	0.36 ± 0.00 ^a^	0.60 ± 0.00 ^d^	0.52 ± 0.02 ^c^

L*, lightness; a*, greenness to redness; b*, blueness to yellowness. Values in the same row with different superscript letters are significantly different (*p* < 0.05).

**Table 2:** Fatty acid composition of samara oil extracted by solvent and mechanical methods (%).

Fatty Acid	Solvent Extraction				Mechanical Extraction	
	Ethyl Acetate	Acetone	*n*-Hexane	Petroleum Ether	Hot-Pressing	Cold-Pressing
C16:0	4.11 ± 0.01 ^a^	4.12 ± 0.02 ^a^	4.10 ± 0.01 ^a^	4.14 ± 0.06 ^a^	4.09 ± 0.00 ^a^	4.12 ± 0.00 ^a^
C18:0	2.61 ± 0.01 ^a^	2.62 ± 0.01 ^a^	2.61 ± 0.03 ^a^	2.65 ± 0.07 ^a^	2.60 ± 0.01 ^a^	2.62 ± 0.02 ^a^
C18:1	36.94 ± 0.13 ^a,b^	36.90 ± 0.06 ^a,b^	36.78 ± 0.14 ^a^	36.96 ± 0.11 ^a,b^	37.14 ± 0.06 ^b^	37.00 ± 0.05 ^a,b^
C18:2 n6	48.28 ± 0.12 ^a^	48.30 ± 0.02 ^a^	48.07 ± 0.26 ^a^	48.26 ± 0.08 ^a^	48.17 ± 0.04 ^a^	48.17 ± 0.02 ^a^
C18:3 n3	7.42 ± 0.03 ^a,b^	7.44 ± 0.01 ^a,b^	7.57 ± 0.15 ^b^	7.37 ± 0.02 ^a^	7.37 ± 0.02 ^a^	7.44 ± 0.01 ^a,b^
C20:0	0.19 ± 0.01 ^a^	0.20 ± 0.00 ^a^	0.18 ± 0.02 ^a^	0.20 ± 0.01 ^a^	0.20 ± 0.00 ^a^	0.20 ± 0.01 ^a^
C20:1	0.45 ± 0.03 ^a^	0.42 ± 0.00 ^a^	0.48 ± 0.00 ^a^	0.42 ± 0.00 ^a^	0.43 ± 0.00 ^a^	0.47 ± 0.03 ^b^
SFA	6.91 ± 0.02 ^a,b^	6.94 ± 0.03 ^b^	6.89 ± 0.02 ^a^	6.99 ± 0.00 ^c^	6.89 ± 0.00 ^a^	6.93 ± 0.00 ^a,b^
MUFA	37.39 ± 0.17 ^a^	37.32 ± 0.06 ^a^	37.46 ± 0.13 ^a^	37.38 ± 0.11 ^a^	37.57 ± 0.06 ^c^	37.47 ± 0.02 ^b^
PUFA	55.70 ± 0.15 ^a^	55.74 ± 0.03 ^a^	55.65 ± 0.11 ^a^	55.63 ± 0.11 ^a^	55.54 ± 0.06 ^a^	55.60 ± 0.03 ^a^

C16:0, palmitic acid; C18:0, stearic acid; C18:1, oleic acid; C18:2n6, linoleic acid; C18:3n3, linolenic acid; C20:0, arachidic acid; C20:1, eicosenoic acid; SFA, saturated fatty acid; MUFA, monounsaturated fatty acid; PUFA, polyunsaturated fatty acid. Values in the same row with different superscript letters are significantly different (*p* < 0.05).

**Table 3:** Bioactive constituents of samara oil extracted by solvent and mechanical methods.

Bioactive Constituents	Solvent Extraction				Mechanical Extraction	
	Ethyl Acetate	Acetone	*n*-Hexane	Petroleum Ether	Hot-Pressing	Cold-Pressing
Polyphenols (mg GAE/kg)	38.01 ± 0.97 ^c^	140.27 ± 26.44 ^d^	24.04 ± 0.39 ^a^	27.88 ± 2.52 ^b^	24.52 ± 0.48 ^a^	23.36 ± 1.94 ^a^
Carotenoids (mg/kg)	5.43 ± 0.33 ^b^	42.95 ± 3.28 ^c^	3.51 ± 0.28 ^a^	3.35 ± 0.33 ^a^	5.03 ± 0.15 ^b^	4.72 ± 0.37 ^b^
γ-tocopherol (mg/kg)	669.98 ± 11.15 ^a^	712.98 ± 9.56 ^c^	688.61 ± 3.74 ^b^	720.10 ± 8.27 ^c^	708.83 ± 18.89 ^c^	722.05 ± 14.11 ^c^

GAE, gallic acid equivalent. Values in the same row with different superscript letters are significantly different (*p* < 0.05).

**Table 4:** Antioxidant activities of samara oil extracted by solvent and mechanical methods.

Parameters	Solvent Extraction				Mechanical Extraction	
	Ethyl Acetate	Acetone	*n*-Hexane	Petroleum Ether	Hot-Pressing	Cold-Pressing
DPPH (μmol TE/100 g)	132.00 ± 1.26 ^d^	817.30 ± 13.73 ^e^	78.50 ± 3.73 ^b^	93.00 ± 11.36 ^c^	56.11 ± 10.04 ^a^	58.12 ± 7.56 ^a^
ABTS (μmol TE/100 g)	628.04 ± 6.11 ^d^	1238.80 ± 3.63 ^e^	561.05 ± 14.66 ^c^	547.90 ± 6.12 ^c^	507.10 ± 8.53 ^b^	450.38 ± 1.22 ^a^
OSI (h)	6.28 ± 0.39 ^a^	21.38 ± 0.53 ^d^	7.75 ± 0.07 ^c^	7.45 ± 0.21 ^b^	7.35 ± 0.28 ^b^	7.35 ± 0.07 ^b^

DPPH, 2,2-diphenyl-1-picrylhydrazy; ABTS, 2,2′-azino-bis(3-ethylbenzothiazoline-6-sulfonic acid) diammonium salt; OSI, oxidative stability index; TE, Trolox equivalent. Values in the same row with different superscript letters are significantly different (*p* < 0.05).

## Data Availability

The data that support the findings of this study are available on request from the corresponding author.
